# Formation and Reactivity of [Au(^13^CN)_4_^(-)^],[Au(^13^CN)_3_Cl^(-)^] and [Au(^13^CN)_3_SR^(-)^] Studied by ^13^C NMR Spectroscopy

**DOI:** 10.1155/MBD.1994.515

**Published:** 1994

**Authors:** Annapurna Jagarlamudi, A. A. Isab, C. Frank Shaw III, A. Munoz

**Affiliations:** 1 Department of Chemistry University of Wisconsin-Milwaukee Milwaukee WI 53201-0413 USA


					FORMATION AND REACTIVITY OF [Au(13CN)4(')], [Au(3CN)3CI(')]
AND [Au(aCN)(SR)I')] STUDIED BY C NMR SPECTROSCOPY
Annapurna Jagarlamudi, A. A. Isab, C. Frank Shaw III and A. Munoz
Department of Chemistry, University of Wisconsin-Milwaukee, Milwaukee, Wl 53201-0413, USA
Au(CN)2- is generated from gold(I) complexes and thiocyanate dtring the oxidative burst.1
Au(CN)2 can be also oxidized to gold(Ill) under similar conditions. Thus, it is of Interest to
examine the chemistry of Au(CN)4-, the gold(Ill), complex of cyanide.
1Ac(13CN)4- can be generated by reacting AuCI4- (yellow, Xmax 316 nm) with 4 equiv, of
N-.12roducing a colourless solution (Xmo, <200 nm). "l'is species is characterized by a
single ];C NMR resonance at 104.6 ppm. In HCI solutions up to 5 M, there is no Initial
displacement of 13CN to form a mixed ligand chloride solution, indicating that the equilibrium lies
far to the left even under drastic conditions:
[Au(CN)4- + HCI < [Au(CN)3CI- + HCN
Upon standing 12-24 hr in 5 or 10 M HCI, however, slow hydrolysis of cyanide to formamide
(HCONH2) drives the formation of [Au(CN)3CI-]"
[Au(CN)4- + HCI + H20 > [Au(CN)3C- + HCONH2
[Au(CN)3CI-] wch is square planar gives a doublet ( 111.1 ppm) and a triplet ( 94.7 ppm),
2:1 ratio, due to zJ13c13C coupling of the cyanides trans and cis to the chlorides.
We also generated [Au(13CN)(SR)] (where SR glutathione, N-acetyI-L-cysteine) by adding one
equivalent of thiol in phosphate buffer. These complexes undergo a slow redox reaction to form
dicyanogold(I) and RSSR:
RSH + [Au(CN)4-
2[Au(CN)3SR-
> [Au(CN)3SR- + HCN
RSSR + [Au(CN)2- + [Au(CN)4-]
Forthionitrobenzoate (TNB) and cysteine the redox reactions apparently proceed faster, so that just
the disulfide and gold(I) cyanide are observed, not the intermediate complex. UV-visible titrations
for the reaction of [Au(CN)4 with excess TNB establish the stoichiometry:
2TNB + [Au(CN)4- > DTNB + [Au(CN)2- + 2HCN
These results suggest that the formation of [Au(CN)4-] can lead to thiol oxidation and re-reduction
of gold(I). Thus, a gold(I)/gold(lll) redox cycle may be established in vivo even under conditions
where generation of gold cyanides from SCN- during the oxidative burst prevails.
G.Graham et aL, Biochem. PharmacoL, 39, 1687-1695 (1990).
C. F. Shaw III et aL, Proc. 3rd Intern. Conf. Gold Silver Med., Metal Based Drugs, this Issue
(1994).
515

				

